# Coherent Tabletop EUV Ptychography of Nanopatterns

**DOI:** 10.1038/s41598-018-34257-2

**Published:** 2018-11-12

**Authors:** Nguyen Xuan Truong, Reza Safaei, Vincent Cardin, Scott M. Lewis, Xiang Li Zhong, François Légaré, Melissa A. Denecke

**Affiliations:** 1 School of Chemistry, The University of Manchester, M13 9PL Manchester, UK; 2 Dalton Nuclear Institute, The University of Manchester, M13 9PL Manchester, UK; 3 INRS, Energie, Matériaux et Télécommunications, 1650 Bld. Lionel Boulet, Varennes, Québec J3X 1S2 Canada; 4 School of Materials, The University of Manchester, M13 9PL Manchester, UK

## Abstract

Coherent diffraction imaging (CDI) or lensless X-ray microscopy has become of great interest for high spatial resolution imaging of, e.g., nanostructures and biological specimens. There is no optics required in between an object and a detector, because the object can be fully recovered from its far-field diffraction pattern with an iterative phase retrieval algorithm. Hence, in principle, a sub-wavelength spatial resolution could be achieved in a high-numerical aperture configuration. With the advances of ultrafast laser technology, high photon flux tabletop Extreme Ultraviolet (EUV) sources based on the high-order harmonic generation (HHG) have become available to small-scale laboratories. In this study, we report on a newly established high photon flux and highly monochromatic 30 nm HHG beamline. Furthermore, we applied ptychography, a scanning CDI version, to probe a nearly periodic nanopattern with the tabletop EUV source. A wide-field view of about 15 × 15 μm was probed with a 2.5 μm−diameter illumination beam at 30 nm. From a set of hundreds of far-field diffraction patterns recorded for different adjacent positions of the object, both the object and the illumination beams were successfully reconstructed with the extended ptychographical iterative engine. By investigating the phase retrieval transfer function, a diffraction-limited resolution of reconstruction of about 32 nm is obtained.

## Introduction

Since the last three decades, coherent diffraction imaging (CDI), also named as lensless X-ray microscopy, has been of great interest as an alternative to the current state-of-the-art microscopy to achieve the atomic-level resolution. Conventional X-ray microscopy often requires multiple extremely precise and pricy optical condensers and deflectors, e.g., Fresnel zone plates or multilayer mirrors, which might introduce optical aberrations or significantly absorb X-rays. Nevertheless, the highest image resolution achieved with X-ray microscopy is about 10 nm^1,2^, which is well far away from the diffraction-limited resolution. As a simple version of X-ray microscopy, CDI is the most efficient way of using photons with the spatial resolution essentially depending only on the wavelength and the highest scattering angle (numerical aperture). In CDI, the exit-surface wave (ESW) diffracted from an object can be fully recovered in both amplitude and phase from a single diffraction pattern (DP) measured in the far-field. According to Sayre^3^, if an experimental DP is sufficiently oversampled, i.e., at least twice the Nyquist frequency, the ESW can be reconstructed with the iterative phase retrieval (IPR) algorithms, giving the physical image of the object. A number of IPR algorithms have been introduced to solve the phase problem for a single experimental DP, such as the error reduction (ER)^4^, hybrid input-output (HIO)^5^, and their modified versions. Basically, an IPR algorithm computes the ESW back and forth between the object- and Fourier-domains, applying certain known constraints such as the Fourier modulus, support, and non-negativity constraints^5,6^. Both ER and HIO algorithms have been widely used in numerous situations^7–15^, but they often suffer from stagnation and trapping in local minima for noisy diffraction patterns^16,17^. Intensive efforts have been made to improve their performance, leading to the introduction of the noise-robust frameworks, e.g., the relaxed averaged alternating reflections (RAAR)^18^, noise-robust HIO^19^, difference-map^20^, oversampling smoothness (OSS)^21^, and optimisation-based IPR algorithms^16,22–24^. Still, CDI works well only for isolated objects. A scanning version of CDI, termed as ptychography, has been proposed for wide-field imaging. In ptychography, multiple well-overlapping areas of an object are sequentially probed with a probe beam and the corresponding (far-field) diffraction patterns are measured. With the additional overlap constraint, ptychography is hence more robust and reliable compared to the conventional CDI, resulting in a higher resolution of reconstruction. Since the first demonstrations in the 1990s^25–28^, ptychography has been increasingly applied to study various kinds of samples including nanostructures and biological cells^29–33^. Recently, for instance, electron ptychographical microscopy has achieved a sub-nm resolution for 3D-imaging of carbon nanotubes^34^. A number of iterative phase retrieval frameworks have been introduced to recover both the object and illumination beam from a ptychographical data set, including the basic and extended ptychographical iterative engines (PIE^35^ and ePIE^36^) and the difference map^30,37^. There exist a few ptychographical solvers such as ptypy^38^, PyNX.Ptycho^39^, and SHARP^40^.

Due to the demand of a great number of coherent X-ray photons, high-resolution ptychography has been often demonstrated at large facilities such as synchrotrons and free electron lasers^41,42^. Thanks to the recent advances in the ultrafast laser technology, coherent tabletop EUV to soft X-ray sources based on the high-order harmonic generation have become achievable in many small-scale laboratories. Future high photon flux and long-term stable HHG sources might offer a unique tool for developing novel phase retrieval algorithms and time-resolved CDI, among other things, in laboratories. So far, only a few groups have been able to demonstrate ptychographical imaging with tabletop EUV sources, mostly at around 30 nm^43–46^ and 13 nm^47,48^. In an attempt to achieve a 100 μm–wide field of view within a single ePIE reconstruction, a polychromatic EUV beam around 29 nm was employed to provide a sufficient photon flux^44^. A breakthrough has been recently made by Gardner *et al*.^47^ in achieving a sub-wavelength spatial resolution for a periodic sample with a 13 nm HHG beam. However, the experimental far-field intensity of the probe beam was required for the image reconstruction^47^, termed as the modulus enforced probe (MEP) approach.

In this study, we demonstrate the preparation of a nanostructured sample by means of the state-of-the-art electron-beam lithography. We then introduce a high photon flux and highly monochromatic tabletop 30 nm beamline, newly established at the Photon Science Institute of the University of Manchester. Finally, we present the first EUV ptychographical imaging of the nearly periodic nanopattern, reconstructed with a modified ePIE without a prior knowledge of the probe.

## Materials and Methods

### Tabletop EUV source

**Figure 1:**
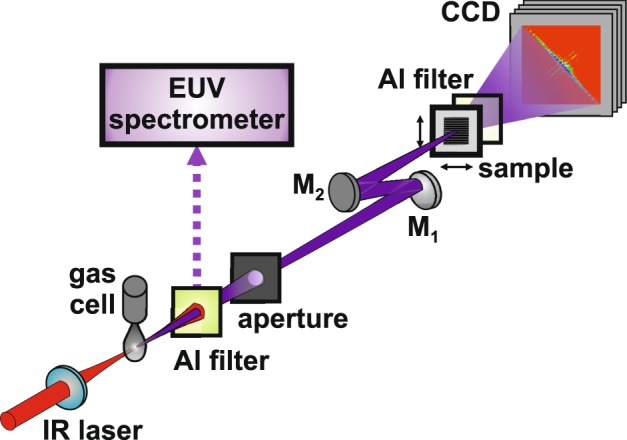
Schematic view of the tabletop EUV source for high-numerical aperture ptychography. An 1 kHz Ti:sa amplifier delivers 35 fs–FWHM optical pulses with up to 8 mJ pulse energy and at an 800 nm central wavelength. The IR beam was focused with a 500 mm focal length lens into an 8 mm long gas cell fed with argon gas through a piezo-driven valve at a backing pressure of 1.5 bars. The resulting HHG beam passed from the source through an 1 mm–inner diameter differential pumping tube to the characterisation chamber, and then was characterised with a flat-field EUV spectrometer. To separate the IR beam, a 300 nm–thick Al foil was used as a spectral filter. At about 80 cm downstream from the HHG source, an 1 mm–diameter aperture was inserted as a spatial filter, resulting in a desired illumination profile for ptychography. A single harmonic at 30 nm was selected and focused with a pair of multilayer mirrors in a z-configuration to minimise astigmatism, including a flat bending mirror (M_1_) and an f_2_ = 110 mm spherical mirror (M_2_). A sample with lithographed nanopatterns was mounted on an xyz-translation stage and located at the focus of the 30 nm probe beam. An SEM image of the sample is provided in Fig. 2a. Diffraction patterns of the adjacent areas on the sample were recorded with an in-vacuum X-ray CCD camera at a distance *z* = 16.5 mm. A second 200 nm–thick Al filter was installed in front of the CCD camera to block the residual stray light. The lateral positioning and data recording were synchronised with a home-built LabVIEW program. Image reconstruction was performed with the extended ptychographical iterative engine (ePIE) on an NVIDIA Tesla K40 computing processor.

The EUV beamline used for ptychography is shown in Fig. 1, including an HHG, a characterisation, and an imaging stage. Great details of the beamline have been given in our recent reports^16,49^. In brief, a femtosecond infrared (IR) Ti:sa laser system provides laser pulses with pulse energy up to 8 mJ, a full-width at half maximum (FWHM) duration of 35 fs and a central wavelength of 800 nm at 1 kHz repetition rate. The IR beam was focused with an f_1_ = 500 mm lens into an 8 mm–long gas cell located in the HHG vacuum chamber. Argon gas was fed into the gas cell with a piezo-driven jet (Attotech) at a backing pressure of 1.5 bars. The gas jet operated at 1 kHz with a typical 250 μs opening time driven by a high-voltage controller. The temporal delay between the IR laser pulses and the gas jet was varied to maximize the HHG yield. The position of the gas cell could be precisely tuned with an xyz-translation and rotation stage. The HHG chamber was separated from the differential pumping chamber by a 100 mm long tube with an 1 mm inner diameter, reducing the gas load in the successive vacuum chambers. The vacuum pressure was ~4 × 10^−3^ mbar in the HHG chamber, and ~10^−6^ mbar in the differential pumping and the experimental chambers. After passing the differential pumping stage, the HHG beam entered the experimental chamber, where it was characterised with a home-built flat-field EUV spectrometer^49^. The IR beam was filtered out with a 300 nm–thick aluminium foil. At about 80 cm downstream from the HHG source, an 1 mm–diameter aperture was inserted as a spatial filter, resulting in a desired EUV beam profile for ptychography.

For ptychographical imaging, a single harmonic at *λ* = 30 nm was selected using a pair of multilayer mirrors (optiX fab) in a z-configuration, containing a flat mirror and an f_2_ = 110 mm mirror. Each mirror has a reflectivity >33% and a FWHM bandwidth of ~1 nm. The fold angles were kept <5° to minimize the astigmatism of the 30 nm probe beam. The spectral bandwidth (λ/∆λ) is about 200, which is sufficient to perform coherent imaging in this work^16,50,51^. A sample with lithographed nanopatterns was mounted on a sub-nm xyz-translation stage (SmarAct) at the focus of the concave multilayer mirror M_2_, perpendicular to the 30 nm probe beam. The light diffracted from the sample was detected with an in-vacuum X-ray CCD camera (Andor iKon-M 934, 1024 × 1024 pixels, and *p*_0_ = 13 µm pixel-size) placed at a distance *z* = 16.5 mm downstream. The CCD’s sensor was cooled down to −95 °C to enhance the signal-to-noise ratio. The camera was rotated an angle *θ* ≈ 45° relative to the sample so that the strongest diffracted signals go along the diagonal of the sensor, optimizing the use of the sensor. A second 200 nm-thick aluminium foil was installed in between the sample and camera to block the residual stray light. By measuring the 30 nm beam profile, a photon flux of ∼7 × 10^7^ photons/s on the detector was obtained. It corresponds to a photon flux of ∼1.1 × 10^8^ photons/s impinging on the sample when taking the absorption of the second Al foil into account. The FWHM-diameter of the focal area of the probe was about *w*_p_ = 2.5 µm, determined by xyz-scanning of a 2 µm–diameter iris and recording the probe beam with the X-ray CCD camera.

### Sample Preparation

Nanopatterns were prepared on a 10 nm thick silicon nitride (Si_3_N_4_) membrane, which acts as an EUV-transmitting window (~15 µm × 15 µm), on a silicon frame (5 mm × 5 mm × 200 µm) by means of the electron beam lithography. To apply the resist to the Si_3_N_4_ window via a spinning process, poly(methyl methacrylate) (PMMA) was used as a bonding agent. The spinning process requires the sample to be held on by a vacuum, which might damage the Si_3_N_4_ membrane. To support the Si_3_N_4_ membrane sample, we first applied a mixture of 8% wt. of anisole and PMMA to the surface of a 10 mm × 10 mm silicon wafer and then placed the sample onto the PMMA. The whole unit was baked on a hot plate at a temperature of 180 °C for 20 minutes, allowing the anisole to evaporate and leaving the PMMA behind. Next, the resist/solvent material was applied to the sample by spin-coating with a speed of 8000 rpm and duration of 40 s. The sample with resist/solvent was then soft baked at a temperature of 100 °C for 2 minutes, resulting in an 100 nm thick resist evenly coated on the sample. Further details are given elsewhere^52^.

**Figure 2:**
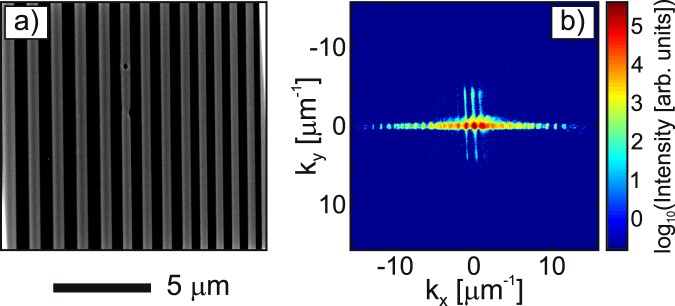
(**a**) An SEM image of the nanopatterned sample. (**b**) A representative diffraction pattern of the sample after binning 2 × 2 pixels into 1 pixel and performing curvature correction. An almost six orders of magnitude high dynamic range image was obtained without the use of a beamstop.

The nanopatterns were written using an FEI scanning electron microscope that was driven by a Raith Elphy Quantum 6 MHz pattern generator. The pattern consisted of boxes that were 50 µm in length and their widths varied from 1 µm to 100 nm, and the line space varied to match the width of the box. The pattern was exposed onto the Si_3_N_4_ window using an acceleration voltage of 30 keV, a current of 50 pA, and a 4 nm step-size. Once the exposure was completed, the sample was developed in hexane for 10 s and the result is shown in Fig. 2a. The final step was to remove the silicon wafer from the sample by dissolving the PMMA bonding agent in an acetone bath for a period of ten hours.

### Image Reconstructions

Image reconstruction was performed with the extended ptychographical iterative engine^36^, including the lateral translation correction^53^. In brief, at the *m*^th^ iteration, a complex-valued probe beam *P*_m_(**r**) illuminates an object *O*_m_(**r**, **R**_j_), coordinated by **R**_j_ as the lateral translation of the object relative to the probe beam. The exit surface wave at the object-plane is given as,

1 $${f}_{{\rm{m}}}({\bf{r}},{{\bf{R}}}_{{\rm{j}}})={P}_{{\rm{m}}}({\bf{r}})\cdot {O}_{{\rm{m}}}({\bf{r}},{{\bf{R}}}_{{\rm{j}}}).$$

At the detector-plane in the far-field, the wavefield is computed as the Fourier transform of the ESW,

2 $${{\bf{F}}}_{{\rm{m}}}({\bf{k}})=FT\{{f}^{{\rm{m}}}({\bf{r}},{{\bf{R}}}_{{\rm{j}}})\}.$$

The modulus constraint replaces the calculated amplitude (|**F**_m_(**K**)|) with the experimental one while maintaining the phase,

3 $${{\bf{F}}}_{{\rm{m}},{\rm{j}}}^{{\rm{mod}}}({\bf{k}})=\sqrt{{I}_{{\rm{j}}}({\bf{k}})}\cdot {{\bf{F}}}_{{\rm{m}}}({\bf{k}})/|{{\bf{F}}}_{{\rm{m}}}({\bf{k}})|.$$

By back-propagating to the object-plane, the modified ESW is then updated as

4 $${f}_{{\rm{m}}}^{{\rm{mod}}}({\bf{r}},{{\bf{R}}}_{{\rm{j}}})=F{T}^{-1}\{{{\bf{F}}}_{{\rm{m}},{\rm{j}}}^{{\rm{mod}}}({\bf{k}})\}.$$

Here, the support constraint might be applied^5,54^. Furthermore, probe and object updates are obtained by applying the overlap constraint, namely,

5 $${O}_{{\rm{m}}+1}({\bf{r}},{{\bf{R}}}_{{\rm{j}}})={O}_{{\rm{m}}}({\bf{r}},{{\bf{R}}}_{{\rm{j}}})+\alpha \frac{{P}_{{\rm{m}}}^{\ast \,}({\bf{r}})}{|{P}_{{\rm{m}}}({\bf{r}}){|}_{{\rm{\max }}}^{2}}[{f}_{{\rm{m}}}^{{\rm{mod}}}({\bf{r}},{{\bf{R}}}_{{\rm{j}}})-{f}_{{\rm{m}}}({\bf{r}},{{\bf{R}}}_{{\rm{j}}})]$$

6 $${P}_{{\rm{m}}+1}({\bf{r}})={P}_{{\rm{m}}}({\bf{r}})+\beta \frac{{O}_{{\rm{m}}}^{\ast \,}({\bf{r}},{{\bf{R}}}_{{\rm{j}}})}{|{O}_{{\rm{m}}}({\bf{r}},{{\bf{R}}}_{{\rm{j}}}){|}_{{\rm{\max }}}^{2}}[{f}_{{\rm{m}}}^{{\rm{mod}}}({\bf{r}},{{\bf{R}}}_{{\rm{j}}})-{f}_{{\rm{m}}}({\bf{r}},{{\bf{R}}}_{{\rm{j}}})],$$

where the empirical parameters (*α*, *β*) are set to unity in this study. The above steps are sequentially repeated for all available scan positions {**R**_j_} to form a single ePIE-iteration. After each complete iteration, to monitor the ePIE progress the normalised error is measured as^36^

7 $${{\rm{\sigma }}}_{m}=\frac{{\sum }_{{\rm{j}}}{\sum }_{{\bf{k}}}{|\sqrt{{I}_{{\rm{j}}}({\bf{k}})}-|{{\bf{F}}}_{{\rm{m}}}({\bf{k}})||}^{2}}{{\sum }_{{\rm{j}}}{\sum }_{{\bf{k}}}{I}_{{\rm{j}}}({\bf{k}})}.$$

Often, the initial probe guess is unity and only updated after a few tens of iterations. Translation correction for each scan position **R**_j_ was performed by calculating the relative shift **δ**_j_(δ_jx_, δ_jy_) between the object’s estimates of the successive iterations, i.e., *O*_m+1_(**r**, **R**j) and *O*_m_(**r**, **R**j), and modifying the current position to

8 $${{\bf{R}}}_{{\rm{j}}}^{{\rm{mod}}}={{\bf{R}}}_{{\rm{j}}}+{\boldsymbol{\gamma }}{{\boldsymbol{\delta }}}_{{\rm{j}}},$$

where the unitless magnification parameter ${\boldsymbol{\gamma }}\,({\gamma }_{x},{\gamma }_{y})$ is a function of the iteration number as suggested in the original work^53^. The lateral shift **δ**_j_ is usually of the sub–0.01 pixel order and was calculated with the cross-correlation technique^53,55^. Due to the invariance of the Fourier-transform to the object’s lateral translation^36,56^, we observed that the translation correction should be always applied (e.g., some tens of iterations) to refine the object and probe functions.

## Results and Discussion

In this study, the sample was raster-scanned with step-sizes (Δ_*x*_, Δ_*y*_) of a few hundreds of nm, i.e., less than *w*_p_/4 to ensure a sufficient (>70%) overlap between the adjacent areas. A random offset of about 15% of the step-sizes was added to each position to avoid the periodic artefacts (known as the *raster grid pathology*)^37^. At each scan position, the diffraction pattern was accumulated three times with 2 s exposure time and an 1 MHz readout speed. Each diffraction pattern was rotated with an angle −*θ* to suit the sample’s coordinates, resulting in a resized image (~1448 × 1448 pixels) with the strongest scattering signals along the x-axis. To improve the signal-to-noise ratio and reduce the computing time, we numerically integrated the diffraction intensity by binning 2 × 2 pixels into 1 pixel and cropped each diffraction pattern to *N* × *N* pixels (with *N* = 600). All diffraction patterns were then remapped onto the Ewald sphere for curvature correction^57,58^, following by a 2D Gaussian smoothing kernel with a standard deviation of 5. A representative diffraction pattern of the sample is shown in Fig. 2b, with the maximum spatial frequency *k*^max^ ≈ 16 μm^−1^. A high dynamic range close to six orders of magnitude was obtained, which is crucial to achieve high-resolution imaging with the ePIE algorithm. The linear oversampling ratio is related to the probe’s diameter as ${{\rm{\Theta }}}_{x}={{\rm{\Theta }}}_{y}=\lambda z/(2{p}_{0}{w}_{p})\approx 7.6$, which is entirely satisfied the oversampling requirement. Note that the effective probe’s beam might be slightly larger than the used FWHM diameter *w*_*p*_, yielding a possible smaller oversampling ratio. In principle, the smallest resolvable period on an object, i.e., the half-pitch distance resolution, is given according to the Rayleigh criterion as, ${\rm{\Delta }}r=\lambda z/(2{p}_{0}N)=31.7$ nm.

In the following, we first explore in details the performance of the modified ePIE framework with a small data set of 169 DPs, probing an area of about 5 × 5 μm of the object. The spatial resolution of reconstruction can be drawn from multiple independent ePIE reconstructions. Second, we present a full field of view of the sample with a data set of 900 DPs, which covers the whole Si_3_N_4_ window.

**Figure 3:**
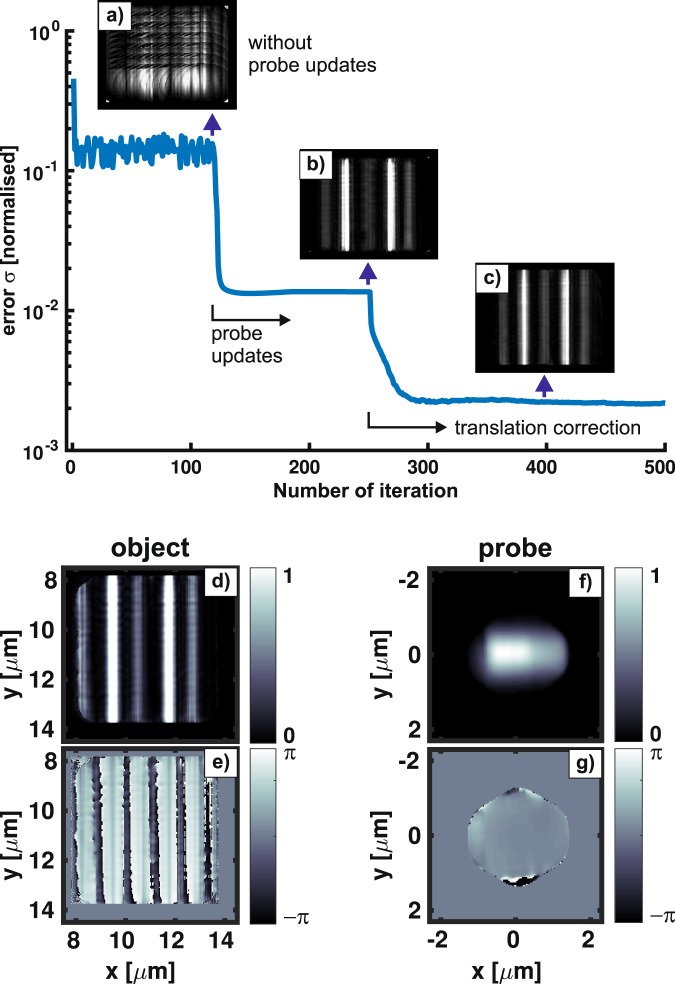
(Top) Typical normalised error as a function of the number of iteration (Eq. (7)). The ePIE algorithm performed 500 iterations with probe updates after 120 iterations and translation refinement after 250 iterations. (Top, insets) The reconstructed images illustrate the visual quality of the object without probe updates (**a**), with probe updates (**b**), and with translation correction (**c**), taken at the iteration marked by vertical arrows. (Bottom) The final reconstructed amplitude and phase of the object (**d**,**e**) and probe (**f**,**g**), respectively.

The first ptychographical scan includes 13 × 13 positions with the step-sizes Δ_*x*_ = Δ_*y*_ = 300 nm. The ePIE started with a probe guess of unity and a random object guess. The ePIE performed 500 iterations with probe updates after 120 iterations and translation refinement after 250 iterations. The ePIE code was written in Matlab (R2016a) and ran on a Tesla K40 GPU accelerator. Figure 3 (top) shows the normalised error as a function of the number of iteration, monitoring the progress of image reconstruction. Snap-shots of the on-the-fly reconstructed object are also depicted to illustrate the improvement (Fig. 3a–c) when different numerical techniques were applied. Essentially, by applying probe update or translation correction, the error metric quickly drops by about an order of magnitude within a few tens of iterations. It then reduces exponentially slow with iteration, representing the characteristics of the well-known ER algorithm built within the ePIE. The final reconstructed amplitude and phase of the object and probe are shown in Fig. 3d–g. The retrieved lines are quite homogenous in the phase’s picture, while their amplitudes are significantly modulated (~40%) from line to line along the x-axis. This effect has been also observed in many other independent reconstructions. We strongly believe that the cross-talk between the object and the probe mainly accounts for the effect. We have performed additional ePIE reconstructions applying the modulus enforced probe approach^47^ and observed much less modulation. However, the corresponding normalised errors are about an order of magnitude higher. The far-field probe’s modulus was measured by moving the sample out of the 30 nm beam.

The spatial resolution can be directly determined if a well-characterised knife-edge sample is available. In CDI, a phase retrieval transfer function (PRTF) has been often used as a powerful mean to gauge the spatial resolution of reconstruction and is given as^14,16,59,60^

9 $${\rm{PRTF}}\,({\bf{k}})=\frac{{|FT\{\langle f({\bf{r}},{{\bf{R}}}_{{\rm{j}}})\rangle \}|}^{2}}{{I}_{{\rm{j}}}({\bf{k}})},$$

where $\langle f({\bf{r}},{{\bf{R}}}_{{\rm{j}}})\rangle$ is the mean ESW at a fixed position **R**_j_, calculated from several independent reconstructions. The resolution cutoff of the ePIE reconstruction can be defined as the spatial frequency at which the PRTF reaches a value of 1/*e* ≈ 0.37. We note, however, that the PRTF might strongly depend on the applied numerical methods^14,16^. Further, care must be taken into account to remove the outliers (failed solutions) before computing the PRTF^61^.

**Figure 4:**
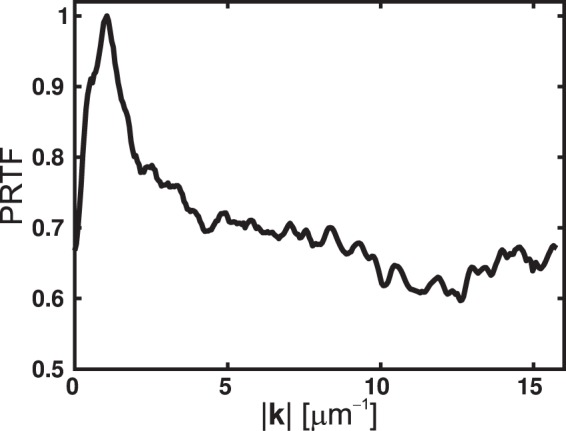
Phase retrieval transfer function (PRTF) computed from five hundred independent ePIE reconstructions. The resolution cutoff of the ePIE, determined by the spatial frequency at which the PRTF reaches a value of 1/*e*, is greater than the experimental cutoff *k*^max^ ≈ 16 μm^−1^.

Figure 4 shows the PRTF obtained from five hundred independent reconstructions with the same setting parameters. Here, the resolution cutoff exceeds the experimental cutoff *k*^max^, which corresponds to the diffraction-limited resolution of 31.7 nm. The resolution of our setup is currently limited by the highest scattering angle recorded with the CCD camera. In addition to using a larger CCD’s sensor, different modifications might be applied to increase the dynamic range of the measured DPs, which is directly related to the highest scattering angle. First, a mechanical beamstop is used to block the brightest undiffracted light, allowing to measure the high-angle diffracted signals with longer exposure time. However, the use of a beamstop often complicates the experimental design and is very time-consuming. Second approach is to stitch different diffraction patterns recorded for various exposure durations, while removing the oversaturated signal^44^. Care must be considered in reading the sensor output, because artefacts (e.g., saturation trail) might occur for oversaturated CCD sensors.

**Figure 5:**
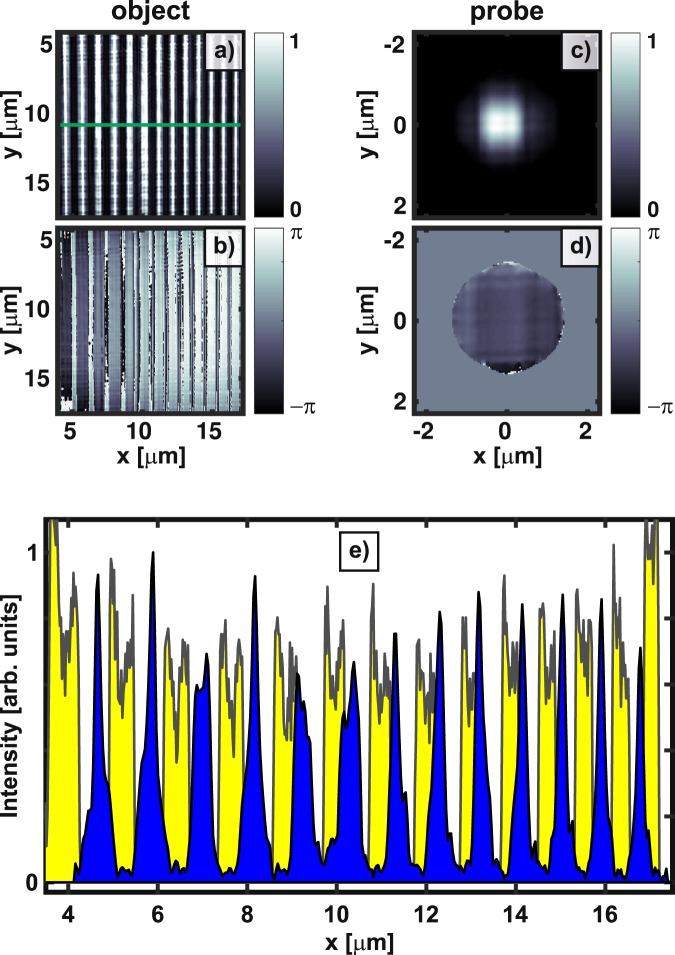
Amplitude and phase of the object (**a**,**b**) and probe (**c**,**d**), respectively, obtained after a thousand ePIE iterations from a collection of 900 far-field diffraction patterns of the sample. (**c**) An intensity line profile (blue-filled) is extracted along the green line in (**a**) compared to its corresponding part of the SEM image (yellow-filled) shown in Fig. 2a. Note the different imaging methods between the ePIE image (transmission) and the SEM (reflection).

For a full-field scan, we consider a ptychographical data set of 30 × 30 positions with the step-sizes Δ_*x*_ = Δ_*y*_ = 400 nm raster-scanning over the whole window (~15 × 15 μm). The ePIE reconstruction performed one thousand iterations with a random object estimate, following with probe updates after 120 iterations, and translation correction after 300 iterations. Figure 5 shows the reconstructed amplitude and phase of the object 5(a,b) and the probe 5(c,d), respectively. A comparison between the ePIE and SEM image is given in Fig. 5e, showing excellent agreement between the two approaches. Note that the ePIE image is obtained in a transmission configuration while the SEM image is obtained in a reflection mode. Compared to the SEM image, the ePIE object shows blurred edges, indicating possible sample’s defects from the e-beam lithography’s preparation. We observe slightly modulated transmission (~15% in average) of the object (5a) along the y-axis with a mean period of about 310 nm, which is probably due to the nearly periodic features of the sample leading to the strong cross-talking between object and probe. In a recent report^47^, this artefact is greatly reduced when the MEP method was applied.

Throughout this work we use for each position a loose support calculated from the inverse FT of the experimental diffraction pattern^6^. We however strongly believe that the visual quality of the object and the progress of image reconstruction might be greatly improved with a dynamic support scheme (e.g., shrink-wrap^54^). The acquisition time of the nine hundred diffraction patterns was about two and half hours. Consequently, we limited ourselves to the thermal drift and mechanical vibrations present in the laboratory. Future developed photon-rich flux EUV sources with high repetition rates (≥100 kHz)^62^ might help to reduce exposure time and enhance the quality of the experimental data.
